# Post-Diverticulitis Colonoscopy Was Not Associated with Higher Colonic Adenoma and Carcinoma: A Multicenter Case–Control Study

**DOI:** 10.3390/medicina57070682

**Published:** 2021-07-02

**Authors:** Amir Mari, Tawfik Khoury, Wisam Sbeit

**Affiliations:** 1 Gastroenterology and Endoscopy United, The Nazareth Hospital, EMMS, Nazareth 16100, Israel; 2 Faculty of Medicine, Bar-Ilan University, Safed 1311502, Israel; tawfikkhoury1@hotmail.com (T.K.); wisams@gmc.gov.il (W.S.); 3 Department of Gastroenterology, Galilee Medical Center, Nahariya 22100, Israel

**Keywords:** diverticulitis, adenomas, CRC, colonoscopy

## Abstract

*Background and Objectives***:** Colonoscopy following an episode of acute diverticulitis is currently recommended to rule out underlying colon cancer. However, a number of studies have debated this recommendation. We aimed to explore whether patients with colonic diverticulosis who experienced an episode of acute diverticulitis had higher prevalence colonic pathologies, essentially colonic adenomas and colorectal carcinoma (CRC) on a follow-up colonoscopy. *Materials and Methods*: We performed a multicenter retrospective study that included patients with a diagnosis diverticulosis as the control group and allocated patients after diverticulitis according to computed tomography (CT) scan and clinical presentation that had performed colonoscopy within 6 months from the acute diverticulitis episode. We compared the detection rate of colonic pathologic findings in both groups. *Results:* Overall, 367 patients were included. Of them, 134 patients experienced an episode of diverticulitis vs. 233 patients who did not have diverticulitis. On univariate analysis, there was no difference between all pathological findings (CRC, colonic adenomas; OR (odds ratio) 1.51, *p* = 0.085), and even for each pathological findings alone, there was no difference (for colonic adenomas, *p* = 0.07; for CRC, *p* = 0.87). Further sub-analysis revealed that only male gender (OR 4.03, *p* = 0.004) and smoking (OR 8.67, *p* < 0.0001) correlated with colonic adenomas and CRC, while moderate to severe disease was not correlated with colonic pathological findings (OR 0.86, 95% CI (confidence interval) 0.4–1.82, *p* = 0.68). *Conclusions:* Post-diverticulitis screening colonoscopy has not found a higher rate of colonic pathological findings, especially colonic neoplasia. Decision to perform colonoscopy after acute diverticulitis should be individualized based on risk stratification of colonic neoplasia.

## 1. Introduction

Colonic diverticulosis is a chronic life-long condition and recently has become one of the most common gastroenterological diseases in western countries, with a global significant healthcare and economic burden [1]. In western populations, more than half of people older than 75 years have diverticulosis [1,2]. Although, generally asymptomatic, diverticulosis may develops complications including acute diverticulitis, bleeding, perforation, and segmental colitis [3]. The incidence of diverticulitis is increasing as demonstrated by a nationwide inpatient study of hospitalizations in the United States showing an increase of 26% in admissions from 1998 to 2005 [4]. Previous studies have reported a high risk of underlying colorectal cancer (CRC) in screening colonoscopy performed for patients following an episode of acute diverticulitis [5,6]. Nonetheless, recent studies have debated these earlier observations [7,8]. Current guidelines of the American Gastroenterology Association recommend diagnostic colonoscopy after resolution of acute diverticulitis to rule out other simulating pathologies such as malignancy, inflammatory bowel disease (IBD), or ischemia [9]. This policy has a major impact on national healthcare services and has led to a high referrals load to endoscopy units as well as producing heavy economic burden. Recent studies have shown that post-diverticulitis colonscopic examination was not associated with increased CRC or colonic adenomas diagnosis [10,11]. Putting everything together and given the uncertainty of this association, we aimed to assess the detection rate of colonic adenomas and CRC by screening colonoscopy after an episode of acute diverticulitis in comparison to patients with diverticulosis but without diverticulitis.

## 2. Materials and Methods

A retrospective case–control study at two Israeli regional academic medical centers (Galilee Medical Center and Nazareth EMMS hospital) was performed. Inclusion criteria included all patients 18 years or older who were admitted to one of the two hospitals from 1 January 2010 till 31 December 2020 and who were diagnosed with diverticulosis or diverticulitis according to computed tomography (CT) scan and had follow-up colonoscopy up to 6 months after that hospitalization. Extracted data included demographic variables (age, gender), background diseases (hyperlipidemia, hypertension, chronic renal failure (CRF), congestive heart failure (CHF), diabetes mellitus, obesity, and smoking), proton pump inhibitor (PPI) use, non-steroidal anti-inflammatory drug (NSAID) use, aspirin and statin use, and colonoscopic findings (site of diverticulosis, and pathological findings on colonoscopy including CRC and colonic adenomas). CRC was defined as a malignant tumor arising from the inner wall of the colon and was confirmed histologically. Colonic adenomas were defined as having polyps with histological diagnosis of tubular adenoma, villous adenomas, and tubulovillous adenoma with either low-grade dysplasia, high-grade dysplasia, carcinoma in situ, or intramucosal carcinoma. The cohort was divided into two groups: patients after an episode of documented acute diverticulitis (cases) vs. patients who only had diverticulosis without diverticulitis (control). Exclusion criteria included patients with concomitant diagnosis of colorectal cancer, inflammatory bowel disease, patients who did not undergo CT scan for diverticulitis confirmation, and patients who did not complete colonoscopy following the episode of diverticulitis. The study protocol conforms to the ethical guidelines of the 1975 Declaration of Helsinki and was approved by the institution human research committee. Written informed consent was waived by the local ethical committee due to the retrospective non-interventional nature of the study.

## 3. Study Endpoints

The primary endpoint of the study was to elucidate whether patients with diverticulosis who had an episode of acute diverticulitis have higher prevalence of CRC and colonic adenomas on post-diverticulitis colonoscopy as compared to patients with uncomplicated asymptomatic diverticulosis. Secondary endpoints were to assess what parameters are associated with the diagnosis of CRC and colonic adenomas. Moreover, we aimed to assess increased diverticulitis severity as being determined by the Hinchey classification using CT scan as follows: Hinchey 0—mild clinical diverticulitis with mild bowel wall inflammation, Hinchey I—localized abscess (para-colonic), Hinchey II—pelvic abscess, Hinchey III—purulent peritonitis (the presence of pus in the abdominal cavity), and Hinchey IV—feculent peritonitis [12], which were associated with higher diagnosis of CRC and colonic adenomas. Patients who presented with Hinchey grade 0 and I were considered as having a mild disease, while patients with Hinchey grades II, III, and IV (generalized fecal peritonitis) were considered to have a moderate to severe complicated diverticulitis.

## 4. Statistical Analysis

Categorical variables were presented as frequencies and percentages as they were analyzed by chi-square test, while continuous variables were reported as mean ± SD using the two-sample *t*-test. A univariate model analysis was performed to assess correlation between pathological colonoscopic findings on colonoscopies in both patient’s groups. Variables with *p*-values less than 0.05 were considered statistically significant. Statistical analyses were carried out using the commercial software Statistical Package for Social Science (SPSS version 24.0, IBM, Chicago, IL, USA).

## 5. Results

### 5.1. Demographics, Baseline Characteristics, and Colonoscopic Findings

**Figure 1:**
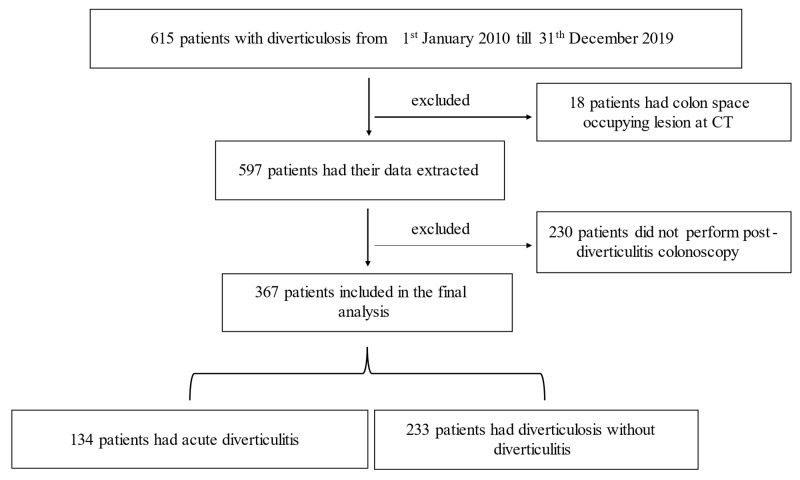
Demonstrate the flow chart of the study cohort.

Overall, 615 patients’ files with confirmed diverticulosis were reviewed; of them, 248 patients were excluded, while the remaining 367 patients were included in the final analysis (Figure 1). Among them, 134 patients had an episode of diverticulitis (group A), as compared to 233 patients who had confirmed diagnosis of diverticulosis without diverticulitis (group B). The average age in group A was 68.3 ± 10.5 as compared to 70.5 ± 7.8 years in group B. Male gender predominated in both groups (69.4% vs. 63.9%, respectively). Only diabetes mellitus and obesity were significantly higher in group A as compared to group B (79.1% vs. 19.3% and 79.1% vs. 18.5%, respectively). Notably, pathological colonoscopic findings including colonic adenomas and CRC did not differ significantly in both groups (32.8% in group A vs. 24.5% in group B, *p* = 0.08). However, when categorizing the pathological findings to include colonic adenomas and CRC, we found that group A was associated with higher detection as compared to group B (32.8% vs. 24.5%, respectively). Table 1 demonstrates the demographics, baselines characteristics, and colonoscopic findings of the study cohort.

### 5.2. Univariate Analysis of the Association between Colonoscopic Findings and Diverticulitis

On univariate analysis, in the primary endpoint, no differences were observed between pathological findings, including CRC and colonic adenomas in both groups (OR 1.51, 95% CI 0.94–2.41, *p* = 0.085). Similarly, there was no difference between each pathological finding alone (for colonic adenomas, *p* = 0.07, and for CRC, *p* = 0.87). Table 2 demonstrates the univariate analysis.

### 5.3. Parameters Associated with the Diagnosis of Colonic Adenomas and CRC among Patients Who Had Diverticulitis

Among patients who had experienced a previous episode of acute diverticulitis, 44 patients had colonic adenomas and CRC (group C), while the other 90 patients had normal colonoscopy (group D). The average age was similar in groups C and D (69.02 ± 9.5 vs. 67.9 ± 10.9, respectively, *p* = 0.27). However, male gender was significantly more common in group C (86.4%) as compared to group D (61.1%) (*p* = 0.001). Notably, there was no difference in the medical history of both groups; however, active smoking status was significantly higher in group C as compared to group D (90.5% vs. 3.4%, respectively; *p* < 0.0001). Table 3 demonstrates the baseline characteristic of the cohort of patients who had experienced an acute diverticulitis. In univariate analysis, male gender (OR 4.03, 95% CI 1.54–10.52, *p* = 0.004) and smoking (OR 8.67, 95% CI 4.54–20.24, *p* < 0.0001) were significantly correlated with colonic findings of adenomas and CRC among patients who had acute diverticulitis.

### 5.4. Association of Diverticulitis Severity with Colonoscopic Diagnosis of Colonic Adenoma and CRC

Overall, there were 50 patients with moderate to severe complicated diverticulitis (Hinchey grades II, III, and IV) who were treated in-hospital, as compared to 84 patients with mild disease (Hinchey grade 0 and I) who were discharged and treated as an outpatient care. There was no difference in the prevalence of colonic adenomas and CRC in the moderate to severe group as compared to the mild disease group (30% vs. 35.5%, respectively, *p* = 0.29). On univariate analysis, pathological findings were not correlated to the diverticulitis severity (OR 0.86, 95% CI 0.4–1.82, *p* = 0.68).

## 6. Discussion

The main finding of our study that patients with diverticulitis did not have more pathological colonoscopic findings (colonic adenomas and CRC) as compared to patients with diverticulosis who did not experience an episode of acute diverticulitis, and there were even no differences when looking separately to CRC and adenomas. Moreover, we found that diverticulitis severity as assessed by Hinchey classification did not correlate with the presence of CRC and adenomas. Nevertheless, our study revealed that among patients who experienced an episode of acute diverticulitis and who had colonic adenomas and CRC, two parameters were significantly associated, including male gender (OR 4.03, 95% CI 1.54–10.52, *p* = 0.004) and smoking (OR 8.67, 95% CI 4.54–20.24, *p* < 0.0001). These observations suggest that in this setting, a screening post-diverticulitis colonoscopy might be considered in male active smokers. To date, professional society guidelines including the American Society of Gastrointestinal Surgery and the American College of Gastroenterology advise performing post-diverticulitis screening colonoscopy to exclude colonic malignancy [13,14]. However, recent studies have shown that the rate of underlying CRC diagnosis in this specific population is very low and probably comparable to the general population undergoing regular screening colonoscopy and suggests its performance in selected patients only [15,16]. One recent study by Khoury and his colleagues reported a very low rate of CRC detection (2 out of 225 patients, 0.89%) in screening post-diverticulitis colonoscopy; furthermore, they reported that male gender was significantly associated with the diagnosis of colonic adenomas and CRC (*p* = 0.039) [11]. Moreover, in concordance with other results, male gender as a risk factor for colonic adenomas and CRC had been addressed by another previous study [17]. Similarly, in our study, we found that male gender was strongly associated with the diagnosis of colorectal neoplasia in screening post-diverticulitis colonoscopy. Expectedly, we found that smoking was also significantly associated with the diagnosis of underlying colonic adenomas and CRC. Our finding was consistent with a previous study by Jung et al. that reported similar results [18]. Moreover, a previous study obviated the need of performing colonoscopy after an episode of diverticulitis in Asian younger patients (<50 years of age) [19]. Given these controversies in this field, a more stepwise approach might be adopted when considering performing a colonoscopy to this population of patients and should be based on the background risk stratification of colonic adenomas and CRC. This approach might be accepted, especially when considering the fact that colonoscopy is associated with potential life-threatening complications in general, and specifically in the setting of diverticulosis as the complication rate including perforation might be higher [20] due to the technical difficulties encountered in diverticular colon such as luminal narrowing, muscular hypertrophy, spasm, and colonic fixation [21,22]. Therefore, this selective more presonalized approach could preclude otherwise well patients from the associated colonoscopy-related risks. Of note, the overall adenoma detection rate (ADR) in our study was 25.9%. This finding is slightly lower than that reported in the literature according to age, as among patients aged up to 50 years, the ADR reached approximately 25%; from age 50–59, the ADR reached approximately 28%; and for those from 60 to 70 years, the ADR reached about 33% [23]. In our study, the average age in both groups was around 69 years, and thus our ADR was slightly lower than that reported according to age groups. Probably this lower rate is due to the inclusion of colonoscopies with non-optimal preparation as well as the presence of diverticulosis. The main limitation of our study is its retrospective nature of data collection. Moreover, another limitation is that we excluded 230 patients as they did not perform post-diverticulitis colonoscopy, which might underestimate the rate of CRC and colonic adenoma, and the last limitation is the inability to include matched control group due to the retrospective design of our study. On the other hand, the strengths were the multicenter nature and the relatively large cohort of patients included.

## 7. Conclusions

To conclude, our study found that screening post-diverticulitis colonoscopy among patients who have experienced an episode of acute diverticulitis was not associated with a higher detection rate of colonic neoplasia diagnosis, while with the presence of male gender and active smoking, colonoscopy is recommended. Therefore, we suggest that the clinical practice of performing follow-up colonoscopy after an episode of acute diverticulitis needs to be individualized and based on potential risks factors for colonic neoplasia, including average risk patients, positive family history of CRC, and patients with pre-existing alarm feature prior to the episode of acute diverticulitis [24]. Finally, a stepwise management approach that takes into consideration the potential harm of missing colonic neoplasia versus the potential adverse events of seemingly unnecessary colonoscopy. Further, large cohort studies are warranted to better understand the place of performing colonoscopy after an episode of acute diverticulitis.

## Figures and Tables

**Table 1:** Demographics, baseline characteristics, and colonoscopic findings.

Parameter	Group A (with Diverticulitis)	Group B (Diverticulosis)	*p*-Value
Number of patients	134	233	-
Age	68.3 ± 10.5	70.5 ± 7.8	0.01
Gender, N (%)			
Male	93 (69.4)	149 (63.9)	0.28
Female	41 (30.6)	84 (36.1)	
Medical history			
Hyperlipidemia	80 (59.7)	136 (58.4)	0.80
Hypertension	97 (72.4)	161 (69.5)	0.51
Chornic renal failure	5 (3.7)	16 (6.9)	0.21
Congestive heart failure	5 (3.7)	15 (6.4)	0.27
Diabetes mellitus	106 (79.1)	45 (19.3)	<0.0001
Obesity	106 (79.1)	43 (18.5)	<0.0001
Smoking, N (%)	43 (31.8)	50 (21.5)	0.02
Statin use, N (%)	62 (46.3)	97 (41.6)	0.38
NSAID use, N (%)	13 (9.7)	19 (8.2)	0.61
Aspirin use, N (%)	81 (60)	127 (54.5)	0.27
Colonoscopic findings, N (%)			
Adenomas and CRC	44 (32.8)	57 (24.5)	0.08
CRC alone	2 (1.5)	4 (1.7)	0.87
Adenomas alone	42 (31.3)	53 (22.7)	0.07
Adenoma detection rate, N (%)	42 (31.3)	53 (22.7)	-

NSAID: non-steroidal anti-inflammatory drug; CRC: colorectal carcinoma.

**Table 2:** Univariate analysis of colonoscopic findings association with diverticulitis.

Pathological Findings	Odds Ratio	95% CI	*p*-Value
Adenomas and CRC	1.51	0.94–2.41	0.085
Adenomas alone	1.60	0.96–2.50	0.07
CRC alone	0.87	0.16–4.80	0.87

CRC: colorectal carcinoma.

**Table 3:** Demographics and baseline characteristics of patients who had acute diverticulitis.

Parameter	Group C	Group D	*p*-Value
Number of patients	44	90	
Age	69.02 ± 9.5	67.9 ± 10.9	0.27
Gender, N (%)			
Male	38 (86.4)	55 (61.1)	0.001 for male
Female	6 (13.6)	35 (38.9)	
Medical history			
Hyperlipidemia	24 (54.5)	56 (62.2)	0.19
Hypertension	31 (70.5)	66 (73.3)	0.36
Chornic renal failure	2 (4.5)	3 (3.3)	0.36
Congestive heart failure	3 (6.8)	2 (2.2)	0.09
Diabetes mellitus	34 (77.3)	72 (80)	0.35
Obesity	34 (77.3)	72 (80)	0.35
Smoking, N (%)	40 (90.5)	3 (3.4)	<0.0001
Statin use, N (%)	21 (47.7)	41 (45.6)	0.40
NSAID use, N (%)	6 (13.6)	7 (7.8)	0.14
Aspirin use, N (%)	28 (63.6)	53 (58.9)	0.30

NSAID: non-steroidal anti-inflammatory drug.

## Data Availability

The data of the study are present at the Gastroenterology department at Galilee Medical Center. The data will be available upon reasonable request.
